# Rapid degeneration of *Drosophila* olfactory neurons in *Orco* mutant maxillary palps

**DOI:** 10.17912/micropub.biology.000398

**Published:** 2021-05-14

**Authors:** Darya Task, Christopher J Potter

**Affiliations:** 1 The Solomon H. Snyder Department of Neuroscience, Johns Hopkins University School of Medicine

## Abstract

*Drosophila melanogaster* vinegar flies have two olfactory organs: the antenna and maxillary palp. Olfactory neurons in these tissues respond to odorants via odorant receptors. Insect odorant receptors are heterotetramers of two proteins: an odorant binding OrX subunit and an Odorant Receptor Co-Receptor (Orco). Mutation of *Orco* disrupts odorant receptor formation, and abolishes olfactory responses. Some antennal olfactory neurons in *Orco* mutants have been previously shown to degenerate. Here, we examine if maxillary palp olfactory neurons also degenerate in *Orco* mutants. We find degeneration occurs both more broadly and more rapidly in *Orco* mutant maxillary palp olfactory neurons than reported for antennae, with ~60% of all mutant olfactory neurons absent in maxillary palps by 7 days post eclosion. Interestingly, the subset of *Orco* mutant olfactory neurons that express the Or42a receptor appear resistant to degeneration. These results suggest the maxillary palp might be a suitable model for examining the molecular mechanisms underlying neurodegeneration in sensory neurons.

**Figure 1. Degeneration of olfactory neurons in Orco mutant maxillary palps:**
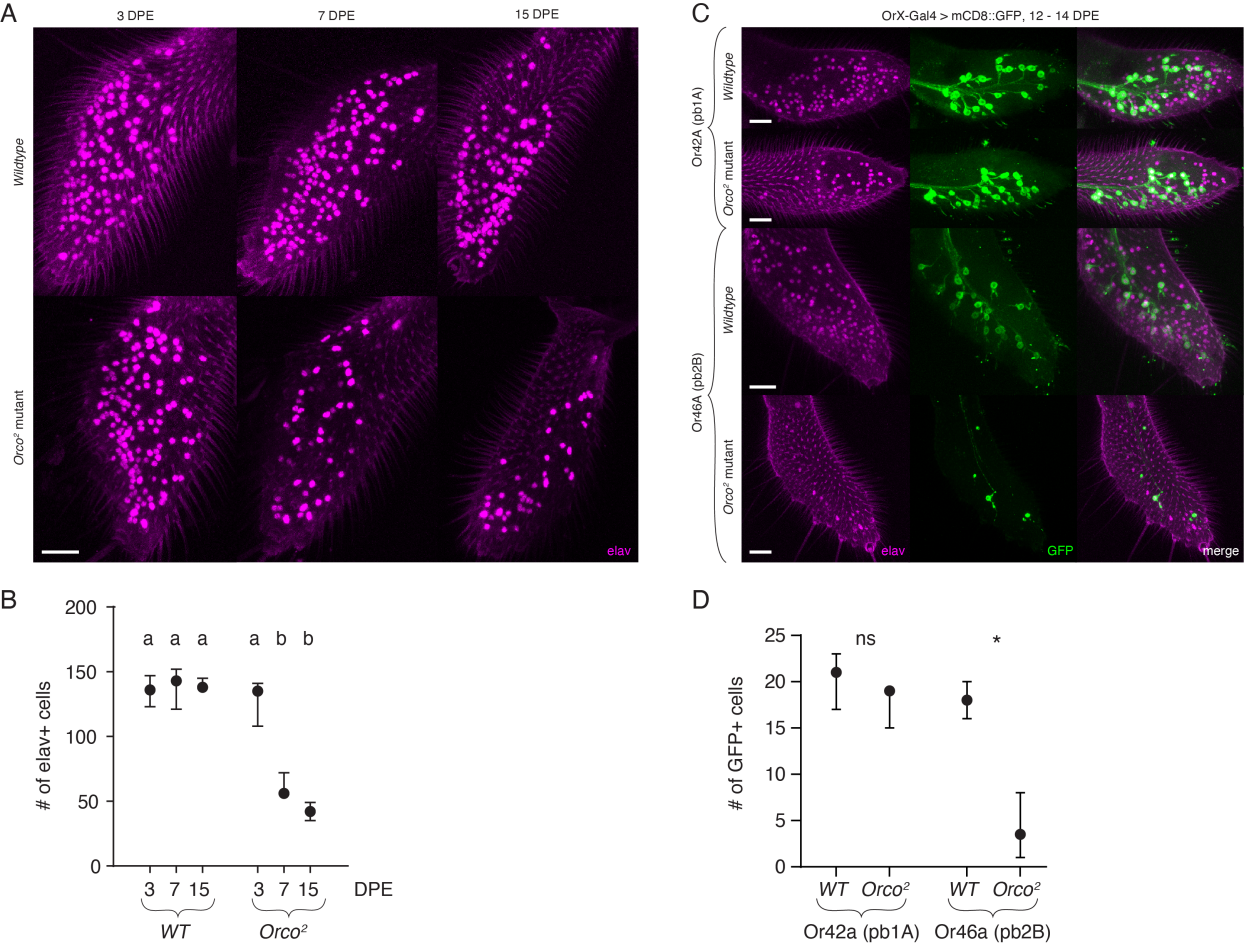
**A.** *Orco^2^* mutant olfactory neuron cell bodies in the maxillary palps are lost by 7 days post eclosion. An anti-elav antibody was used to visualize all neuronal cell bodies in the palps. This includes all olfactory neurons (~120), as well as a small population of presumed mechanosensory neurons (~20). *Wildtype* (top row) and *Orco^2^* mutant (bottom row) palps compared at three timepoints: 3 (left column), 7 (middle column), and 15 (right column) days post eclosion (DPE). **B.** Quantification of elav+ cells in *wildtype* and *Orco^2 ^*mutant palps at the three timepoints (N = 3 per condition). *Wildtype* flies have consistent numbers of neurons throughout. *Orco^2 ^*mutant palps appear normal at 3 DPE but lose approximately 60% of their neurons by day 7. At 15 DPE the *Orco^2 ^*mutant phenotype is not significantly different from 7 DPE. There was a statistically significant difference between groups as determined by an one-way ANOVA (*F*(5,12) = 40.54, *p* < 0.0001). A Tukey’s post-hoc test for all pairwise comparisons revealed a statistically significant difference of the two oldest *Orco^2^* mutant timepoints (7 and 15 DPE) from all other conditions (denoted with letter b; *p* ≤ 0.0002), but no significant difference between these two conditions (p = 0.4055). There was no statistically significant difference between any of the *wildtype* conditions (p > 0.99), nor between the 3 DPE *Orco^2^* mutant and any of the *wildtype* timepoints (*p* > 0.79) (denoted with letter a). **C.** Subsets of *Orco* mutant olfactory neurons avoid degeneration. The *GAL4*/*UAS* system was used to GFP label pb1A (Or42a+) or pb2B (Or46a+) olfactory neurons in *wildtype* or *Orco^2^* mutant palps. All flies were 12 – 14 DPE. **D.** Quantification of GFP+ pb1A and pb2B olfactory neurons in *wildtype* and *Orco^2^* mutant palps. Mann-Whitney *U* tests showed a statistically significant difference between *wildtype* and *Orco^2^* mutant in pb2B neurons (*Mdn_OrcoMut_* = 3.5, *Mdn_wildtype_* = 18.0, *U*(*N_OrcoMut_* = 4, *N_wildtype_* = 4) = 0, *p* = 0.0286), denoted by an asterisk, but not in pb1A neurons (*Mdn_OrcoMut_* = 19.0, *Mdn_wildtype_* = 21.0, *U*(*N_OrcoMut_* = 3, *N_wildtype_* = 4) = 2, *p* = 0.2000), denoted by ns (not significant), indicating that the latter neurons are largely spared (N = 3-4 per condition). Graphed in (B) and (D) are medians with 95% confidence intervals. Scale bars in (A) and (C) are 20 µm.

## Description

The odorant receptor co-receptor Orco forms ligand-gated ion channels with odorant receptors (ORs) tuned to specific odors; Orco is required for trafficking of these complexes to the dendrites of olfactory neurons (Larsson et. al., 2004). Mutation of the *Orco* gene not only abolishes olfactory neuron activity but has also revealed potential new roles for this gene in the development and maintenance of olfactory systems in a variety of insects. In ants and honeybees, *Orco* mutation leads to a loss of olfactory sensory neurons in the periphery, as well as severe defects in the morphology of the antennal lobe, the first olfactory processing center in the insect brain (Trible *et al.*, 2017; Yan *et al.*, 2017; Chen *et al.*, 2021). These deficits are likely developmental as they are apparent at adult eclosion. *Orco* mutant hawkmoth males show more modest losses in antennal lobe volume associated with pheromone-sensing olfactory neurons (Fandino *et al.*, 2019). In contrast, in *Anopheles coluzzii* and *Aedes aegypti* mosquitoes *Orco* mutation does not appear to lead to olfactory neuron loss or gross deficits in antennal lobe anatomy (though the latter has not been examined in depth) (Sun *et al.*, 2020; DeGennaro *et al.*, 2013).

In the vinegar fly *Drosophila melanogaster*, development of the adult olfactory system in *Orco* mutants appears normal (Larsson et. al., 2004). However, over time some populations of olfactory neurons in the antennae degenerate. This degeneration is initially evident in the olfactory neuron axons innervating the antennal lobe, which start to show signs of blebbing and retraction four to six days post eclosion (DPE) (Chiang *et al.*, 2009). By 14 DPE, some but not all antennal olfactory neurons lose their cell bodies (Hueston *et al.*, 2016). There is evidence that the cause of antennal olfactory neuron degeneration in the *Orco* mutant is due to a lack of neuronal activity (Chiang *et al.*, 2009). Here, we investigated if neurodegeneration in *Orco* mutants also occurs in the *Drosophila* maxillary palp, a simpler olfactory organ with ~120 olfactory neurons.

To examine the effects of the loss of *Orco* on maxillary palp olfactory neurons, we performed whole-mount antibody staining on maxillary palps using the pan-neuronal marker, *elav* (Robinow and White, 1991). This marker should label all ~120 olfactory neurons in the palps, as well as a small population (~20) of presumed mechanosensory neurons (Singh and Nayak, 1985). Neuronal cell death should result in a reduction in the number of elav+ cells. Potential alternative approaches could be to use DAPI staining or the TUNEL assay to visualize cell loss and DNA fragmentation associated with cell death, among others (Meehan *et al.*, 2015). We compared anti-elav antibody staining in *wildtype* and *Orco^2^* mutant flies at three timepoints: 3, 7, and 15 days post eclosion (DPE) (**Fig 1A**). In the *wildtype*, we found consistent neuronal cell counts across timepoints. The *Orco^2^* mutant palps of 3-day-old flies were indistinguishable from *wildtype* (**Fig 1A**). However, by 15 DPE, extensive loss of cell bodies was detectable in the *Orco^2^* mutants (**Fig 1A**). This neurodegeneration was evident at 7 DPE (middle column), suggesting that cell body loss in the maxillary palp occurs more quickly and potentially more broadly than in the antennae. Overall, *Orco^2^* mutants lose 60 – 70% of their maxillary palp neurons. These results are quantified in **Fig** **1B**. Both 7 and 15 DPE *Orco^2^* mutants had significantly fewer elav+ cells compared to all *wildtype* timepoints as well as 3 DPE *Orco^2^* mutants (*p* ≤ 0.0002) but were not statistically significantly different from each other (*p* = 0.4055) (one-way ANOVA with Tukey’s HSD; see figure legend for *F* statistic and exact *p* values for all pairwise comparisons). There was no difference between any of the *wildtype* conditions (*p* > 0.99), nor between the 3 DPE *Orco^2^* mutant and any of the *wildtype* timepoints (*p* > 0.79).

We further examined if sub-populations of olfactory neurons in the maxillary palp might be resistant to degeneration. There are six different populations of olfactory neurons in the maxillary palp as defined by the odor-responsive OrX they express. Using available genetic reagents, we examined neuron degeneration in two of these populations in the *Orco* mutant background. We used the *GAL4*/*UAS* system (Brand and Perrimon, 1993) to GFP label the pb1A (*Or42a-GAL4*/*UAS-mCD8:GFP*) or pb2B (*Or46a-GAL4*/*UAS-mCD8:GFP*) olfactory neurons (Goldman *et al.*, 2005) in *wildtype* or *Orco^2^* mutant maxillary palps. We examined flies 12 – 14 DPE to allow sufficient time for degeneration. Surprisingly, we found that the pb1A (Or42a-expressing) olfactory neuron population was largely spared from degeneration (**Fig 1C,** quantified in **Fig 1D**). In contrast, the pb2B (Or46a-expressing) olfactory neurons were almost all missing by 14 DPE (**Fig 1C**, quantified in **Fig 1D**). Those pb2B cells that remained showed signs of neuronal process fragmentation and blebbing, consistent with previously reported antennal neuron degeneration (Chiang *et al.*, 2009). Prior work has confirmed that both these populations express *Orco* (Grabe *et al.*, 2016; Larsson *et al.*, 2004; Task *et al.*, 2020). Our results suggest that at least one sub-population of *Orco* mutant olfactory neurons are resistant to activity-dependent cell death and represents the first quantified example of a specific olfactory neuron population escaping degeneration. While pb1A cells are presumably non-functional due to a lack of *Orco*, future experiments will be required to confirm that this is indeed the case, especially in light of recent studies showing co-expression of odorant receptors with other, non-*Orco*-dependent chemoreceptors in the same cells (McLaughlin *et al.*, 2021; Task *et al.*, 2020; Younger and Herre *et al.*, 2020). This could be achieved by genetically silencing pb1A activity, as has previously been done in the antenna (Chiang *et al.*, 2009). Interestingly, Orco-dependent cell death in the antennae does not involve caspase-dependent pathways and cannot be rescued by expression of the pan-caspase inhibitor p35 (Chiang *et al.*, 2009). It remains to be determined if *Orco* mutant neurons in the maxillary palp similarly engage caspase-independent molecular mechanisms.

The olfactory system of insects represents a convenient model for examining how a lack of induced activity might affect the health of a sensory neuron (MacDonald *et al.*, 2006; Chiang *et al.*, 2009; Kazama *et al.*, 2011; Hueston *et al.*, 2016). As shown here, the maxillary palp presents a potentially favorable system for studying neurodegeneration and activity-dependent neuronal maintenance. In contrast to the antenna, neuronal cell body loss in *Orco* mutant palps is rapid (within 6 or 7 days post eclosion vs. 14 days post eclosion in antennae). The palp is a simpler olfactory organ, and neuronal changes are easier to study and quantify by whole-mount staining in the palps. We present evidence that at least one genetically definable population of olfactory neurons appears resistant to degeneration. Future experiments could examine if any of the other four olfactory sub-populations in the maxillary palp also demonstrate such resistance. Given the extensive genetic tools available in the *Drosophila* model system, maxillary palp olfactory neurons might be amenable to genetic screens aimed at investigating the molecular mechanisms underlying activity-dependent sensory degeneration.

## Methods

Fly husbandry and *Drosophila* genetics

Fly stocks were maintained at 20 – 25°C on standard cornmeal-agar food. Male and female flies used for experiments were 3 – 15 days old. Exact age for each experimental condition indicated in **Figure 1**. Experimental flies were homozygous for *Orco^2^*, while control flies were either *w^1118^* wildtypes (**Figure 1A-B**), or heterozygous for *Orco^2^* (*Orco^2^*/*TM6b*; **Figure 1C-D**). Full genotypes of stocks used in **Figure 1C-D**: *Or42a-Gal4, 10XUAS-IVS-mCD8::GFP / CyO ; Orco^2^ / TM6b* and *Or46a-Gal4, 10XUAS-IVS-mCD8::GFP / CyO ; Orco^2^ / TM6b*.

Immunohistochemistry

All immunostaining steps were done while rotating. Fly proboscises (labella and palps) were dissected in 1XPBS and fixed in 4% paraformaldehyde in PBT (1XPBS + 0.3% Triton X-100) for 15 minutes at room temperature. Tissue was washed three times for 15 minutes each at room temperature in PBT, then blocked for at least 30 minutes at room temperature in blocking solution (PBT + 5% normal goat serum). Primary antibodies were added to fresh blocking solution, and tissue was incubated in this solution overnight at 4°C. On day two, tissue was washed three times for 15 minutes each at room temperature in PBT, then incubated in secondary antibodies in fresh block overnight at 4°C in the dark. On day three, tissue was washed three times for 15 minutes each at room temperature in the dark and mounted in SlowFade Gold (ThermoFisher S36936). Palps were dissected from labella on the slide before mounting. Primary antibodies were used at 1:100 concentration, secondary antibodies were used at 1:200 concentration. See Reagents for antibodies used.

Confocal imaging and analysis

Palps were imaged on a Zeiss LSM 700 confocal microscope equipped with a C-Apochromat 63x/1.2 water Korr M27 objective. Images were acquired at 512 x 512-pixel resolution with 0.58 µm z-step. For illustration purposes, confocal images were processed in Fiji/ImageJ to collapse Z-stacks into a single image using maximum intensity projection. Fiji was also used to adjust the gain in separate channels; no other image processing was performed on the confocal data. Elav+ and GFP+ cells were counted manually in Fiji using the Cell Counter plugin.

Statistics

All statistical analyses and plots were done in GraphPad Prism (version 8). For all analyses, significance level α = 0.05. In **Figure 1A-B**, one-way ANOVA with Tukey’s HSD post-hoc test was used to compare elav+ cell counts across both genotypes at the three timepoints. In **Figure 1C-D**, Mann Whitney *U* tests were used to compare GFP+ cell counts in the two genotypes within each neuron type.

## Reagents

*Drosophila melanogaster* stocks used:

**Table:** 

**Genotype**	**Source**	**Identifier**
w[*]; P{w[+mC]=Or42a-GAL4.F}48.3B	Bloomington *Drosophila* Stock Center	BDSC: 9970; FlyBase: FBti0101811
*w[1118]; P(w[+mC]=Or46a-GAL4.G)32.1.y*	Bloomington *Drosophila* Stock Center	BDSC: 23291; FlyBase: FBti0076800
w[*]; P(y[+t7.7] w[+mC]=10XUAS-IVS-mCD8::GFP)attP40	Bloomington *Drosophila* Stock Center	BDSC: 32186; FlyBase: FBti0131963
*Orco^2^* mutant*:* w[*]; TI(w[+m*]=TI)Orco[2]	Bloomington *Drosophila* Stock Center	BDSC: 23130; FlyBase: FBti0168777
Wildtype: *w^1118^ IsoD1*	Gift from Thomas R. Clandinin	Derived from FBal0018186
Double Balancer: *y,w; Pin/CyO; Dh/TM6B*	Potter lab stock	Derived from FBal0013831, FBba0000025, FBti0004009, FBba0000057, FBal0016730
Double Balancer: *y,w; S/CyO; Pr/TM6B*	Potter lab stock	Derived from FBal0015108, FBba0000025, FBal0013944, FBba0000057, FBal0016730

Antibodies used:

**Table:** 

**Antibody**	**Source**	**Identifier**
Rat anti-elav	DSHB	Cat# Rat-Elav-7E8A10; RRID: AB_528218
Chicken anti-GFP	Aves Labs	Cat# GFP-1010; RRID: AB_2307313
Goat anti-rat Cy3	Jackson ImmunoResearch	Cat# 112-165-167; RRID: AB_2338251
Goat anti-rat Alexa 647	Jackson ImmunoResearch	Cat# 112-605-167; RRID: AB_2338404
Goat anti-chicken Alexa 488	Invitrogen	Cat# A11039; RRID: AB_142924
